# Anti-*N*-methyl-D-aspartate receptor encephalitis: the clinical course in light of the chemokine and cytokine levels in cerebrospinal fluid

**DOI:** 10.1186/s12974-016-0507-9

**Published:** 2016-03-03

**Authors:** Zuzana Liba, Jana Kayserova, Martin Elisak, Petr Marusic, Hana Nohejlova, Jitka Hanzalova, Vladimir Komarek, Anna Sediva

**Affiliations:** Department of Pediatric Neurology, 2nd Faculty of Medicine, Charles University and Motol University Hospital, V Uvalu 84, Prague, 15006 Czech Republic; Department of Immunology, 2nd Faculty of Medicine, Charles University and Motol University Hospital, Prague, Czech Republic; Department of Neurology, 2nd Faculty of Medicine, Charles University and Motol University Hospital, Prague, Czech Republic

**Keywords:** Anti-*N*-methyl-D-aspartate receptor encephalitis, T cell, Cytokines, CXCL10 chemokine, CXCL13 chemokine

## Abstract

**Background:**

Anti-*N*-methyl-D-aspartate receptor (NMDAR) encephalitis is an autoimmune disorder of the central nervous system (CNS). Its immunopathogenesis has been proposed to include early cerebrospinal fluid (CSF) lymphocytosis, subsequent CNS disease restriction and B cell mechanism predominance. There are limited data regarding T cell involvement in the disease. To contribute to the current knowledge, we investigated the complex system of chemokines and cytokines related to B and T cell functions in CSF and sera samples from anti-NMDAR encephalitis patients at different time-points of the disease. One patient in our study group had a long-persisting coma and underwent extraordinary immunosuppressive therapy.

**Methods:**

Twenty-seven paired CSF/serum samples were collected from nine patients during the follow-up period (median 12 months, range 1–26 months). The patient samples were stratified into three periods after the onset of the first disease symptom and compared with the controls. Modified Rankin score (mRS) defined the clinical status. The concentrations of the chemokines (C-X-C motif ligand (CXCL)10, CXCL8 and C-C motif ligand 2 (CCL2)) and the cytokines (interferon (IFN)γ, interleukin (IL)4, IL7, IL15, IL17A and tumour necrosis factor (TNF)α) were measured with Luminex multiple bead technology. The B cell-activating factor (BAFF) and CXCL13 concentrations were determined via enzyme-linked immunosorbent assay. We correlated the disease period with the mRS, pleocytosis and the levels of all of the investigated chemokines and cytokines. Non-parametric tests were used, a *P* value <0.05 was considered to be significant.

**Results:**

The increased CXCL10 and CXCL13 CSF levels accompanied early-stage disease progression and pleocytosis. The CSF CXCL10 and CXCL13 levels were the highest in the most complicated patient. The CSF BAFF levels remained unchanged through the periods. In contrast, the CSF levels of T cell-related cytokines (INFγ, TNFα and IL17A) and IL15 were slightly increased at all of the periods examined. No dynamic changes in chemokine and cytokine levels were observed in the peripheral blood.

**Conclusions:**

Our data support the hypothesis that anti-NMDAR encephalitis is restricted to the CNS and that chemoattraction of immune cells dominates at its early stage. Furthermore, our findings raise the question of whether T cells are involved in this disease.

**Electronic supplementary material:**

The online version of this article (doi:10.1186/s12974-016-0507-9) contains supplementary material, which is available to authorized users.

## Background

Anti-*N*-methyl-D-aspartate receptor (NMDAR) encephalitis is an autoimmune disorder of the central nervous system (CNS) that predominantly affects young females [1]. The progressive multistage development of psychiatric and neurologic symptoms represents a typical clinical manifestation of the disease [2]. This clinical entity was first reported in 2007 in a group of women with ovarian teratomas and paraneoplastic production of anti-NMDAR antibodies [3]. Subsequently, it was demonstrated that this clinical phenotype arises from antibody-mediated internalization of NMDARs and that non-paraneoplastic production of anti-NMDAR antibodies is also possible [4].

The immunopathogenesis of this disease has yet to be fully elucidated, although B cells, but not T cells, have been proposed to be involved [5]. Based on clinical observation, a model of early cerebrospinal fluid (CSF) lymphocytosis and subsequent expansion of the immune repertoire within the intrathecal compartment was suggested [6], but immunological data supporting this model are insufficient.

To contribute to the current knowledge regarding the immune mechanisms that participate in anti-NMDAR encephalitis, we studied the complex system of chemokines and cytokines in CSF and sera from anti-NMDAR encephalitis patients at different time-points of the disease. Chemokines and cytokines are produced by various cell types and reflect complex immune processes. Chemokines attract immune cells to sites of action and, thus, might be responsible for the initial pleocytosis. We selected the following chemokines: C-C motif ligand 2 (CCL2) for monocytes, C-X-C motif ligand 8 (CXCL8) for neutrophils, CXCL10 for T cells and CXCL13 for B cells. B cell-activating factor (BAFF) was measured as a critical marker of B cell activation and survival. Based on the understanding that T helper (Th) cells might support B cell functions [7], we measured the levels of cytokines that are associated with Th cell function, including interferon γ (IFNγ), tumour necrosis factor α (TNFα), interleukin (IL)4 and IL17A [8]. Additionally, the cytokine profile was determined by evaluating the levels of IL2, IL7 and IL15, which participate in the differentiation and survival of different subsets of lymphocytes, particularly T cells [9].

## Methods

### Subjects

Nine Caucasian patients (age: median 13 years, range 7–26 years; sex: 8 females, 1 male) with non-paraneoplastic anti-NMDAR encephalitis were included in this study. The patients were diagnosed and/or treated at the Motol University Hospital, Prague, Czech Republic.

**Fig. 1:**
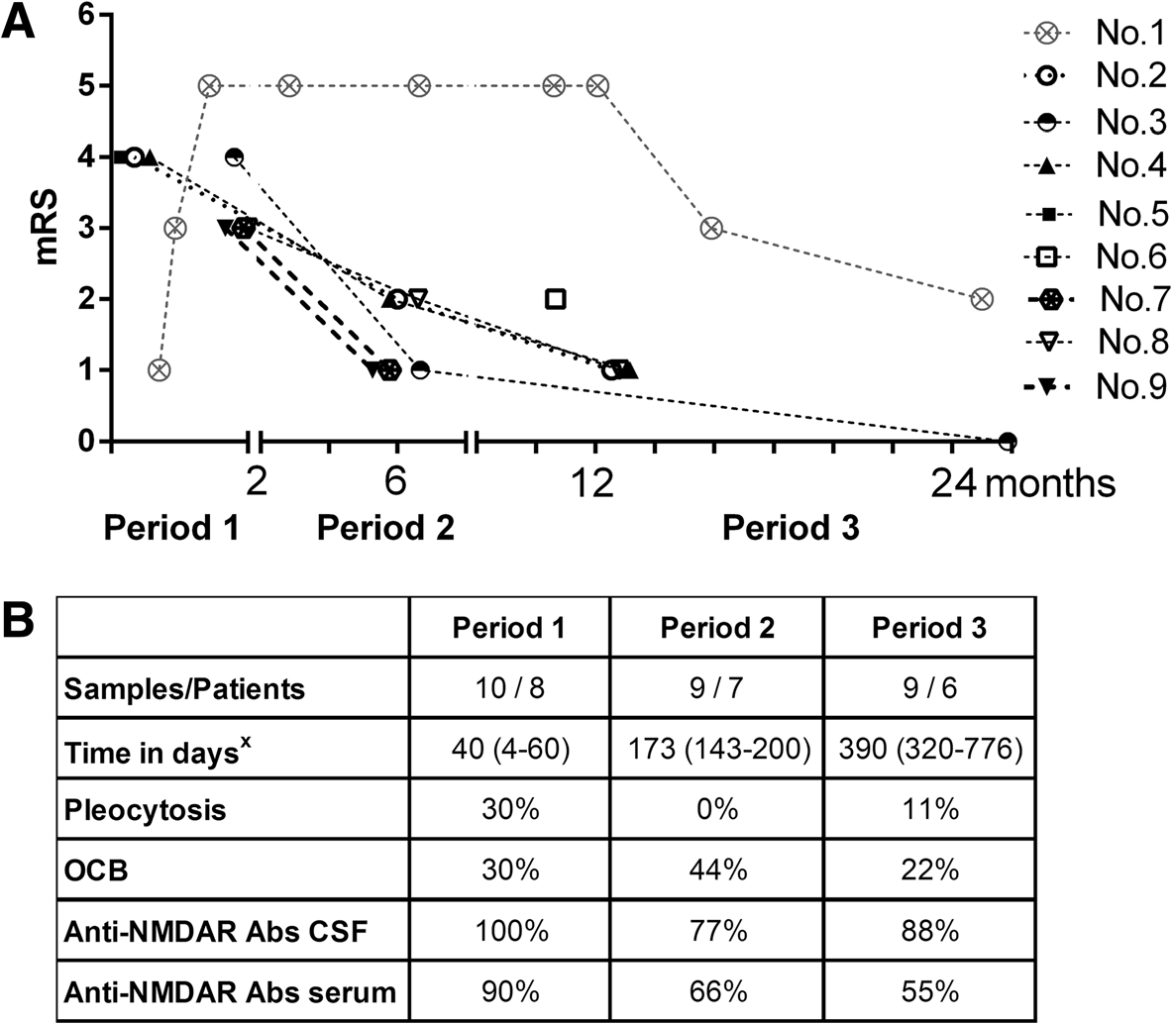
Stratification of CSF samples and the laboratory data. **a** For 7/9 patients with anti-NMDAR encephalitis, two or more CSF and serum samples were collected at different time-points. In total, we collected 27 paired CSF and serum samples from the patients during the 26-month observation period. The clinical status (as defined by the mRS) was assigned to every sample, and the successive samples from the same patient are marked. All of the patients except for no. 8 and no. 9 were treatment naïve in period 1. Patients nos. 1, 8 and 9 did not respond sufficiently to the first-line immunotherapy, and the second-line treatment was planned. All of the patients in period 2 were already treated. Patient no. 6 was diagnosed late; she was the only treatment naïve patient in period 3. **b** The samples from the patients were stratified into three periods (periods 1–3) after the appearance of the first disease symptom ^x^(days; median and range). The following common laboratory data are summarized in the table (percentage among all of the samples in the given period): pleocytosis in CSF (as defined by leukocytes > 5/μL); oligoclonal bands (OCB) in CSF; and anti-NMDAR antibody (Abs) positivity in CSF and serum (defined qualitatively)

The patients’ CSF and sera were positive for anti-NMDAR antibodies at the time of diagnosis. Their clinical status was defined using the modified Rankin score (mRS). Magnetic resonance imaging of the brain, electrophysiological studies and oncological assessments were performed upon disease diagnosis, and the oncological assessments were repeated every 6 months. Seven of the nine patients underwent more than one CSF and serum withdrawal during the follow-up period (median 12 months, range 5–26 months) (Fig. 1a).

The control group consisted of ten Caucasian patients (age: median 12 years, range 8–18 years; sex: 8 females, 2 males) who underwent a single CSF and serum withdrawal due to the following symptoms: headache (*n* = 5), visual impairment (*n* = 2), anxiety (*n* = 2) or tics (*n* = 1). The findings in CSF and sera from the controls were within the normal range, and no anti-NMDAR antibodies were detected.

This study was approved by the Ethical Committee at the Motol University Hospital. Informed consent was obtained from all participants.

### Samples

In total, 27 paired CSF/serum samples from nine patients and ten paired CSF/serum samples from ten controls were tested. The patient samples were stratified into three periods after the appearance of the first disease symptom: *period 1* (eight patients; time-point of sample collection: median 40 days, range 4–60 days); *period 2* (seven patients; time-point of sample collection: median 173 days, range 143–200 days) and *period 3* (six patients; time-point of sample collection: median 390 days, range 320–776 days). Stratification of the samples according to the mRS and the laboratory results is shown in Fig. 1a, b.

Anti-NMDAR antibodies in CSF and sera were detected using commercial kits (anti-glutamate receptor [type NMDA] IIFT, Euroimmun, Germany). This cell-based assay enabled the qualitative detection of anti-NMDAR antibodies in the samples; quantitative analysis was not performed.

The routine analysis of CSF included determinations of the following: cell count, protein levels, CSF/serum albumin ratio, immunoglobulin (Ig)G and IgM levels and oligoclonal band levels.

Aliquots of centrifuged CSF and serum samples were immediately stored at −20 °C and thawed once before use for chemokine and cytokine analysis.

### Chemokine and cytokine detection

The concentrations of the chemokines CCL2, CXCL8 and CXCL10 and the cytokines IFNγ, IL2, IL4, IL7, IL15, IL17A and TNFα were measured with Luminex multiple bead technology according to the manufacturer’s instructions (ProcartaPlex Human Simplex Immunoassay, eBioscience, San Diego, CA, USA). The data were collected using the Luminex-100 system (Luminex, Austin, TX, USA). The BAFF and CXCL13 concentrations were determined via enzyme-linked immunosorbent assay according to the manufacturer’s instructions using software from R&D Systems (Minneapolis, MN, USA).

### Data analysis and statistics

Statistical analyses were performed using GraphPad PRISM, version 6.0 (GraphPad Software, La Jolla, CA, USA). Non-parametric tests were used. The Wilcoxon signed-rank test was used for pairwise comparisons of the CSF and sera samples. The Kruskal-Wallis test was performed to compare multiple groups of samples, and Dunn’s multiple comparisons test was employed for post hoc analysis. Patient no. 1 provided multiple samples at different time-points in each period, and we used the average values in each period for multiple-group comparisons. The correlations between the parameters were calculated using the Spearman correlation. A *P* value <0.05 was considered to be significant.

## Results

### Clinical data

The clinical peak of the disease manifested within 30 days of the first disease symptom onset in all of the patients (median 23 days, range 6-30 days). Nevertheless, the disease severity varied among the patients. Notably, patient nos. 1 and 6 differed markedly from the others (Fig. 1a). Data from patient no. 1 are depicted separately in the text. Patient no. 6 manifested only moderate symptoms of the disease and was diagnosed and treated with a delay.

The recovery time lasted for months. The mRS significantly decreased in period 2 (median mRS = 2) compared to period 1 (median mRS = 4, *P* = 0.0006, Mann-Whitney *U* test). Clinical improvement continued into period 3 (median mRS = 1), although this result was not significant. Generally, the patients reached mRS ≤2 in a median time of 3 months (range 1–25 months).

The therapies were initiated within 60 days of disease onset in all cases except for patient no. 6 (median 21 days, range 6–320 days). The patients were treated as recommended [1]. Intravenous steroids, plasma exchange or high-dose intravenous immunoglobulins were the first treatment choices. Three patients did not respond sufficiently to the first-line immunotherapy and received a second-line treatment (nos. 1, 8 and 9). Patient no. 1 remained in a coma and was treated with a monoclonal antibody against CD52 (alemtuzumab) and with two doses of intrathecal methotrexate followed by oral mycophenolate mophetil (Fig. 3a) [10]. None of the patients relapsed. The patient data are summarized in Table 1.

**Table 1:** Patient data

Patients									
Number	1	2	3	4	5	6	7	8	9
Sex	F	F	F	F	M	F	F	F	F
Age of onset (years)	7	10	13	13	13	18	20	18	26
Follow-up (months)	25	12	26	13	1	1	6	12	5
Clinical features at the disease peak									
Psychiatric features	Yes	Yes	Yes	Yes	Yes	Yes	Yes	Yes	Yes
Cognitive dysfunction	Yes	Yes	Yes	Yes	Yes	Yes	Yes	Yes	Yes
Seizures	Yes	No	Yes	Yes	No	Yes	Yes	Yes	Yes
Movement disorder	Yes	Yes	No	Yes	Yes	No	No	Yes	No
Autonomic dysfunction	Yes	No	No	No	No	No	No	Yes	No
Coma	Yes	No	No	No	No	No	No	Yes	No
Laboratory features at the disease diagnosis									
MRI changes	No	No	No	Yes	No	No	No	No	Yes
EEG changes	Yes	Yes	Yes	Yes	Yes	Yes	Yes	Yes	Yes
Disease course and therapy									
Progression (days)^a^	28	15	28	17	6	16	30	29	29
Time to therapy (days)^b^	6	15	49	21	4	320	60	21	29
Recovery (days)^c^	750	43	91	72	30	320	36	150	85
Therapy^d^	IVIG, CS, PE, RTX, CPA, IA, alemtuzumab, MTX, MMF	PE, CS, IVIG	PE, CS, IVIG	PE, CS, IVIG	CS, IVIG	PE, IVIG, CS	CS, IVIG	PE, IVIG, CS, RTX	PE, CS, CPA

This table summarizes the clinical, laboratory and follow-up data of nine patients with non-paraneoplastic anti-NMDAR encephalitis who were included in this study. Our clinical study was focused on the dynamic course of the disease over time (in days after the onset of the first disease symptom): ^a^time to the peak in disease severity (all of the patients reached their disease severity peak within 30 days, and despite receiving immunotherapy, patients nos. 1 and 8 progressed to a mRS of 5); ^b^time to first therapy administration (all of the patients except for patient no. 6 received therapy within 60 days); and ^c^time to clinical improvement to a mRS ≤2 (the recovery time varied between the patients: median 85 days, range 30–750 days; patient no. 6 had a mRS of 2 at the time of diagnosis). ^d^The therapy summary is indicated as follows: *CPA* cyclophosphamide, *CS* corticosteroids, *IA* immunoadsorption, *MMF* mycophenolate mophetil, *MTX* methotrexate, *PE* plasma exchange, *RTX* rituximab

### Chemokine and cytokine assays

**Fig. 2:**
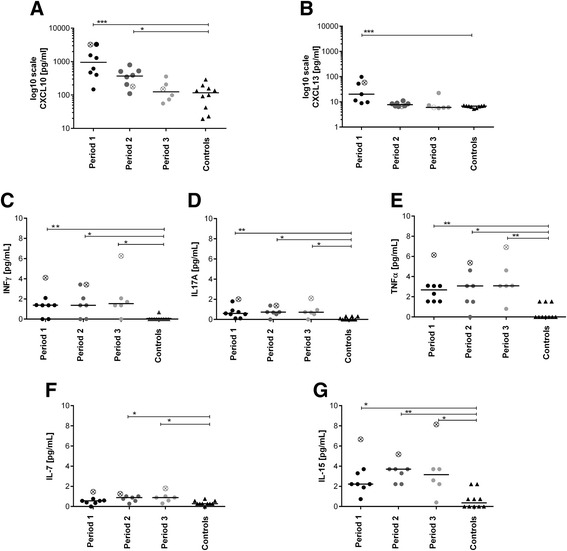
Chemokine and cytokine levels in CSF. The patient CSF chemokine and cytokine levels in each of the three previously defined periods were compared with the corresponding control levels. The Kruskal-Wallis test (K-W) was used for multiple-group comparisons, and Dunn’s test was employed for post hoc analysis (statistical significance is denoted as *P < 0.05, **P < 0.005 or ***P < 0.0005). Each sample in each period belonged to one patient. Because patient no. 1 provided more than one sample per period, the average value for this patient was used for multiple-group analysis; a special sign marks this value. Chemokines: **a** Increased CXCL10 levels were observed during periods 1 and 2 (K-W: 0.0005; Dunn’s: *P* < 0.0005 and *P* < 0.05, respectively); **b** The CXCL13 level was increased only in period 1 (K-W: 0.0015; Dunn’s: *P* < 0.0005). Th cell-related cytokines: **c** The IFNγ, **d** TNFα and **e** IL17A levels were significantly increased in the patients at all periods during the follow-up period compared with the controls (K-W IFNγ: 0.02, K-W TNFα: 0.02, K-W IL17A: 0.004; Dunn’s: *P* < 0.05, details provided in the figure). Cytokines important for T cell survival and function: **f** Increased IL7 levels were detected in periods 2 and 3 (K-W: 0.017; Dunn’s: *P* < 0.05). **g** A significant increase in the IL15 levels were observed at all periods (K-W: 0.0013; Dunn’s: *P* < 0.05, details are provided in the figure)

We analysed the complex system of chemokines and cytokines in patient CSF and serum samples at three previously defined periods compared to corresponding control samples. Samples from period 1 were obtained from 6/8 treatment naïve patients. The remaining samples from period 1 were obtained from patients who responded insufficiently to the first-line immunotherapy, and the second-line treatment was planned (nos. 8 and 9). Samples in periods 2 and 3 were from treated patients. Only one sample in period 3 was from treatment naïve patient (no. 6). Marked changes in the levels of chemokines and cytokines were observed in the CNS compartment but not in the peripheral blood (Fig. 2a–g). Important overall differences between the CNS compartment and the peripheral blood are summarized in Additional file 1: Figure S1A–C.

### Chemokine levels in CSF

The CSF CXCL10 and CXCL13 concentrations were significantly increased during period 1 and the CXCL10 level remained significantly increased during period 2 in the patients compared with the controls (Fig. 2a, b). This cross-sectional study did not reveal any significant differences in the CXCL8 or CCL2 levels (data not shown).

### T and B cell-related cytokines in CSF

*INFγ*, *TNFα* and *IL17A*: The CSF levels of these Th cell-related cytokines were significantly increased at all periods in the patients compared with the controls (Fig. 1c–e). *IL7* and *IL15*: The CSF IL15 concentration remained also significantly increased at all periods in the patients compared with the controls (Fig. 2g). The CSF IL7 concentration was slightly yet significantly increased during periods 2 and 3 in the patients compared with the controls (Fig. 2f). *BAFF*, *IL2*, and *IL4*: This longitudinal study did not reveal any significant differences in the CSF BAFF concentration. The CSF IL2 and IL4 concentrations were generally below their respective detection limits (data not shown).

### Correlation of the investigated parameters in anti-NMDAR encephalitis

We correlated the disease period with the clinical status (mRS), pleocytosis and the levels of all of the investigated chemokines and cytokines. Correlations between time and the mRS (*r* = −0.41, *P* = 0.04) and the CSF leukocyte numbers (*r* = −0.46, *P* = 0.02) and chemokine levels (CXCL10: *r* = −0.68, *P* = 0.0001; CXCL13: *r* = −0.75, *P* < 0.0001) were observed. Additionally, CSF pleocytosis correlated with the chemokine levels (CXCL10: *r* = 0.47, *P* = 0.02; CXCL13: *r* = 0.40, *P* = 0.047, data not shown). Time did not correlate with the levels of B or T cell-related cytokines (BAFF, TNFα, IFNγ, IL17A, IL7 and IL15).

### Single case study

**Fig. 3:**
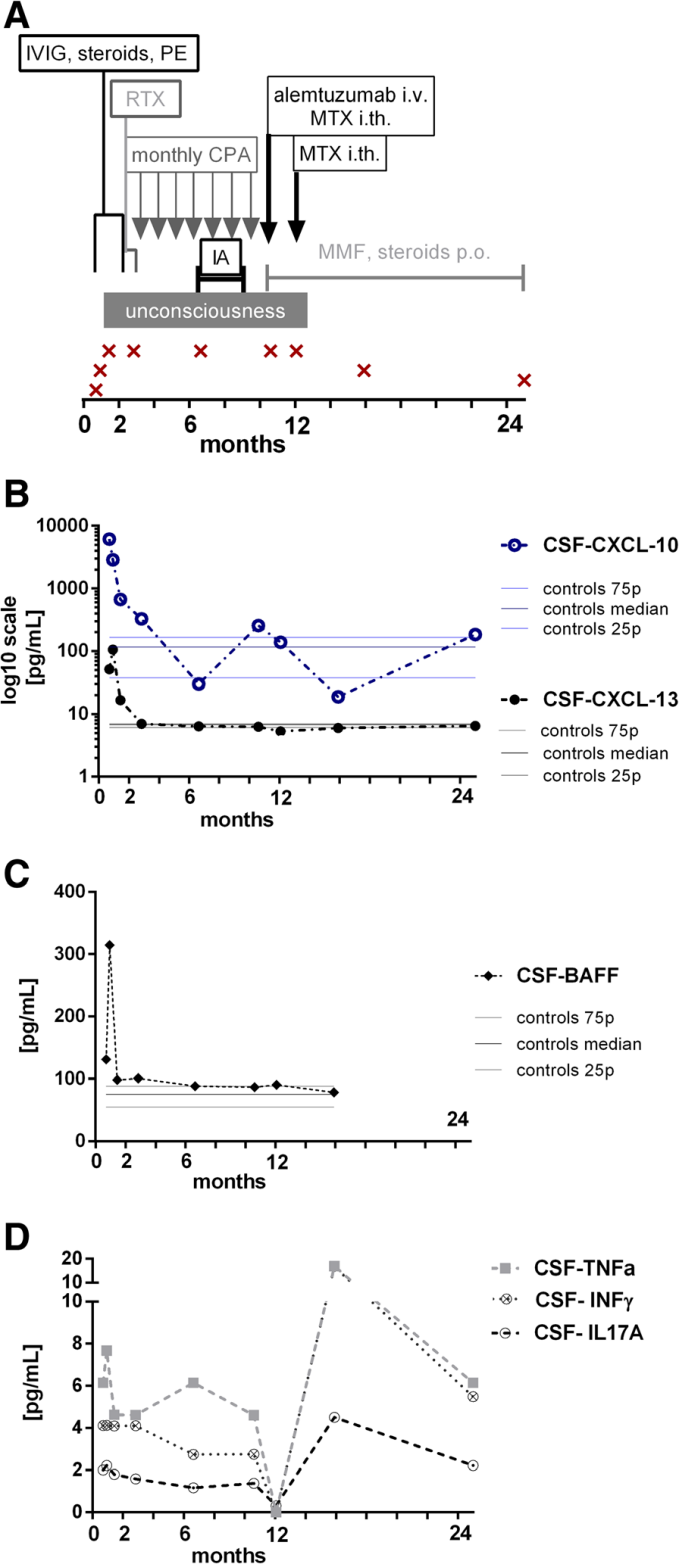
Single case study. A 7-year-old girl represented the most complicated patient in the group, and she remained in a coma for 344 days. **a** The timeline (in months) of combined immunotherapy and sample withdrawal is shown. She did not respond to the first- or second-line immunotherapy (immunoglobulins = IVIG, plasma exchange = PE, immunoadsorption = IA, rituximab = RTX, cyclophosphamide = CPA). Then, she was experimentally treated with intravenous alemtuzumab combined with two doses of intrathecal methotrexate (MTX) followed by oral mycophenolate mophetil (MMF). Nine successive CSF samples were collected during the 25-month follow-up period. The first two samples anticipated the rapid clinical deterioration (mRS = 1 and 3). The five subsequent samples were collected while she was in a coma (mRS = 5), and the final two samples were collected during the recovery period (mRS = 3 and 2). **b** The CSF levels of the chemokines CXCL10 and CXCL13 and **c** of BAFF peaked at disease onset (period 1). The increase in the CXCL13 levels preceded the increase in the CXC10 levels and was followed by rapid clinical deterioration. Subsequently, the concentrations of these factors remained unremarkable despite her coma, as illustrated by the interquartile ranges of the levels in the controls. **d** In contrast, the CSF levels of Th cell-related cytokines (IFNγ, TNFα and IL17A) were increased throughout the follow-up period. The exception was observed in the sample after the first dose of intrathecal methotrexate and intravenous alemtuzumab, which was shortly before her clinical improvement. Then, the levels of these cytokines increased again without any signs of clinical relapse. The interquartile range of the levels of these cytokines in the controls did not exceed 1.2 pg/mL

Patient no. 1, a 7-year-old girl, represented a minor group of patients that do not respond to common therapy guidelines and are in a high risk of death [1]. Despite an early immunotherapy, the disease progression lasted 28 days, and subsequently, she remained in a coma for 344 days. Finally, she recovered when a biological treatment that depleted B and T cell populations in peripheral blood was administered in combination with a cytostatic agent intrathecally (Fig. 3a). We evaluated nine paired CSF and serum samples from this patient, which were collected over a 25-month follow-up period (Fig. 1a).

The initial three CSF samples reflected her rapid clinical deterioration into a coma (mRS 1, 3 and 5). In particular, pleocytosis progression (7/μL and 77/μL, respectively) and markedly increased levels of all of the investigated chemokines and cytokines were observed in the first two samples. Interestingly, the increase in the CXCL10 levels preceded the increase in the CXCL13 and BAFF levels, and these concentrations were the highest among all patients. Pleocytosis was not observed, and the chemokine and BAFF levels were not increased in the five subsequent samples that were collected during her coma (Fig. 3b, c). In contrast, the increases in the CSF levels of T cell-related cytokines (INFγ, TNFα and IL17A), IL7 and IL15 persisted through the whole follow-up period (period 1–3, Fig. 2c–g). A transitory decrease in T cell-related cytokine levels was observed after the intrathecal application of methotrexate, shortly before her clinical improvement (Fig. 3d).

## Discussion

Immune mechanisms in anti-NMDAR encephalitis are currently being intensively studied. Analysis of chemokine and cytokine levels in a clinical context can contribute to a better understanding of the immune processes this disease. Our study was focused on the complex spectrum of chemokines and cytokines related to B and T cell functions and their changes over the different clinical courses of anti-NMDAR encephalitis. To our knowledge, no other cytokine study in this disease has been published except the one by Leypoldt and his colleagues [11].

The role of B cells in anti-NMDAR encephalitis has already been demonstrated [5, 12, 13]; however, the mechanism underlying their entry into the CNS compartment is unclear. Moreover, the quantity of B cells is scarce as T cells predominate in the CSF of healthy individuals [14]. CXCL13 was designated as the major determinant for B cell recruitment to the CNS compartment during neuroinflammation [15, 16]. We observed lower CXCL13 levels in the CNS compartment than in the peripheral blood; however, these levels were increased in the patients compared with the controls (Additional file 1: Figure S1A). This chemokine binds to C-X-C receptor 5 (CXCR5) and reflects an antibody-mediated immunopathology [17]. Alternatively, CXCL10 binds to CXCR3, which is expressed on different subsets of T cells, including memory Th1 cells [18, 19]. Notably, IFNγ induces CXCR3 expression on antibody-producing memory B cells [20] and integrates with an increased CXCL10 level to promote the stimulation of B cells in the CNS compartment during viral encephalomyelitis [21].

We showed marked changes in the CSF levels of T and B cell-related chemokines (CXCL10 and CXCL13). The significantly increased levels of these chemokines were observed at the early stage of the disease when the clinical symptoms were the most prominent (period 1, Fig. 2a, b). Furthermore, these chemokine levels correlated with CSF pleocytosis. Interestingly, the initial CXCL10 and CXCL13 levels were the highest in the most complicated patient during her rapid clinical deterioration (Fig. 3b). During the following periods, these chemokine levels decreased (periods 2 and 3, Fig. 2a, b).

CXCL13 was recently proposed as a biomarker of the treatment response in anti-NMDAR encephalitis [11]. Our findings regarding CXCL10 and CXCL13 raised the question of whether the initial levels of these chemokines could be potential predictive biomarkers of disease severity.

B cells are expected to thrive in the CNS compartment due to the BAFF levels [22], which were previously studied in other B cell-associated CNS pathologies [23, 24]. Despite this expectation, no significant differences in the BAFF concentrations were detected in our study. The CSF BAFF levels in patient no. 1 did not differ from those in the other patients, except for an isolated peak, which preceded her coma (Fig. 3c).

Further, we hypothesized that T cells could be involved in the disease immunopathogenesis. It was recently reported that Th17 cells appear to be more efficient in supporting B cell responses outside the germinal centres and in promoting antibody production than their Th1 counterparts based on animal models [25]. IL17A was previously studied in antibody-mediated CNS disorders, such as neuromyelitis optica [26]. We investigated a set of specific cytokines that might reflect the involvement of T cells in anti-NMDAR encephalitis. Indeed, we observed a slight yet significant increase in the CSF levels of INFγ, TNFα, IL17A, IL7 and IL15 in these patients. These Th1-/Th17-related cytokines and the crucial cytokine for T cell survival did not correlate with time and remained elevated throughout the periods examined. Notably, the highest levels of these cytokines were observed in the samples of the most complicated patient (periods 1–3, Fig. 2c–g). The relevance of these findings is currently unclear and warrants careful follow-up of patients who are recovering from this disease.

## Conclusions

Our investigation of chemokines and cytokines in the CSF and sera of anti-NMDAR encephalitis patients supported the hypothesis that the disease immunopathology is restricted to the CNS compartment. The dynamics of CXCL10 and CXCL13 CSF levels further supported the hypothesis that chemoattraction of the immune cells dominates at the early stage of the disease. Our findings regarding CXCL10, IFNγ, TNFα, IL17A and IL15 raise the question of possible involvement of T cells in this disease. The role of these cytokines in anti-NMDAR encephalitis remains to be elucidated.

## Additional file

Additional file 1: Figure S1. Chemokine and cytokine levels in CSF and peripheral blood. The Wilcoxon signed ranked test was used for pairwise analyses of the CSF and sera samples to detect differences between the CNS compartment and the peripheral blood. The interesting differences in the general chemokine and cytokine levels between the CNS compartment and the peripheral blood are depicted. A) The CXCL10 concentration was markedly higher in CSF than in serum, whereas B) the CXCL13 and D) BAFF concentrations showed the opposite trend. Because patient No. 1 provided more than 1 sample per period, the average value for this patient was used for multiple-group analysis; a special sign marks this value. (TIF 79 kb)

## Abbreviations

BAFF: B cell-activating factor
CCL2/MCP1: monocyte chemotactic protein 1/C-C motif ligand 2 chemokine
CXCL: C-X-C motif ligand chemokine
CXCL10/IP-10: IFNγ-inducible protein 10/lymphocyte-specific C-X-C chemokine
CXCL13/BCA1: B cell-attracting chemokine 1
CXCL8/IL-8: neutrophil chemotactic factor
IFN: interferon
IL: interleukin
mRS: modified Rankin score
NMDAR: N-methyl-d-aspartate receptor
TNF: tumour necrosis factor
